# Virulence Genes of Pathogenic *Escherichia coli* in Wild Red Foxes (*Vulpes vulpes*)

**DOI:** 10.3390/ani12151959

**Published:** 2022-08-02

**Authors:** Fabrizio Bertelloni, Giulia Cagnoli, Fabrizio Biagini, Alessandro Poli, Carlo Bibbiani, Valentina Virginia Ebani

**Affiliations:** 1 Department of Veterinary Sciences, University of Pisa, Viale delle Piagge 2, 56124 Pisa, Italy; fabrizio.bertelloni@unipi.it (F.B.); giulia.cagnoli@vet.unipi.it (G.C.); fabriziobiagini3@gmail.com (F.B.); alessandro.poli@unipi.it (A.P.); carlo.bibbiani@unipi.it (C.B.); 2 Centre for Climate Change Impact, University of Pisa, Via del Borghetto 80, 56124 Pisa, Italy

**Keywords:** *Escherichia coli*, virulence genes, pathotypes, STEC, *Vulpes vulpes*

## Abstract

**Simple Summary:**

*Escherichia coli* is a commensal of the intestinal tract of humans and animals, but some pathotypes can cause severe infections. Enteropathogenic *E. coli* (EPEC), Shiga toxin-producing *E. coli* (STEC), and enterohemorrhagic *E. coli* (EHEC) are the pathotypes most frequently involved in enteric disorders observed in people and domestic animals. Wildlife may harbor and excrete these pathotypes too, therefore, they may be source of infections for humans and domestic animals. *Vulpes vulpes* seem to be involved in the epidemiology of pathogenic *E. coli* strains, and thus they could be a relevant threat mainly when they invade human settlements in rural and urban areas.

**Abstract:**

Different pathotypes of *Escherichia coli* can cause severe diseases in animals and humans. Wildlife may contribute to the circulation of pathogenic pathotypes, including enteropathogenic *E. coli* (EPEC), Shiga toxin-producing *E. coli* (STEC), and enterohemorrhagic *E. coli* (EHEC). This study analyzed 109 DNA samples previously extracted from fecal specimens collected from red foxes (*Vulpes vulpes*) to detect *E. coli* virulence genes *eaeA*, *hlyA*, *stx1*, and *stx2*, that characterize the EPEC, STEC, and EHEC strains. Thirty-one (28.4%) samples were positive for at least one investigated virulence gene: *eaeA* gene was detected in 21 (19.2%) samples, *hlyA* in 10 (9.1%), *stx1* in 6 (5.5%), and *stx2* in 4 (3.6%). Nine DNA samples resulted positive for two or three virulence genes: five (4.6%) samples were positive for *eaeA* and *hlyA* genes, two (1.8%) for *eaeA* and *stx1*, one (0.9%) for *hlyA* and *stx1*, one (0.9%) for *eaeA*, *hlyA* and *stx2*. Red foxes seem to be involved in the epidemiology of these infections and their role could be relevant because they may be source of pathogenic *E. coli* for other wild animals, as well as domestic animals and humans.

## 1. Introduction

*Escherichia coli*, family Enterobacteriaceae, is an opportunistic gram-negative bacterium commensal of the human and animal intestinal tract. Extra-intestinal infections, mainly urinary tract infection, meningitis, and septicemia, due to *E. coli* strains are frequent threats in human and veterinary medicine [1]. Enteric forms caused by diarrhoeagenic *E. coli* strains are often observed too. These forms are related to different *E. coli* pathotypes acting with different mechanisms in relation to their virulence traits. In particular, enteropathogenic *E. coli* (EPEC), Shiga toxin-producing *E. coli* (STEC), and its subgroup of enterohemorrhagic *E. coli* (EHEC) are the pathotypes most frequently involved in enteric disorders of humans and other animals [2].

EPEC strains produce intimin, an adherence factor encoded by the *eae* gene found at the locus of enterocyte effacement (LEE), embedded on a large chromosomal pathogenicity island (PAI) [3]. These strains are able to adhere to enterocytes inducing microvilli loss and consequent diarrhea [4].

STEC are characterized by the production of two types of Shiga toxins (Stx1 and Stx2), encoded by lambdoid bacteriophages, which are maintained in lysogeny in the bacterial chromosome [5]. Stx1 and Stx2, that include several subtypes, are known as two of the most potent bacterial toxins; they exhibit differences in cytotoxicity to various cell types, bind dissimilarly to receptor analogs or mimics, induce differential chemokine responses, and have several distinctive structural characteristics. Their action in the intestinal tract are very important being toxic to colonic, ileal epithelial, and endothelial cells. Stx1 is 98% homologous to the Stx produced by *Shigella dysenteriae* type 1, while Stx2 is about 60% homologous with Stx1 and is antigenically different [6,7].

EHEC are STEC strains have intimin and hemolysin, among their virulence factors. Hemolysin, encoded by *hly* gene, contributes to the pathogenesis by different mechanisms, such as hemolysis, the induction of pro-inflammatory reactions, and epithelial and endothelial cells damages [8]. STEC and EHEC cause different clinical manifestations in humans, including asymptomatic carriage, bloody or severe diarrhea, and systemic diseases, such as hemorrhagic colitis (HC) and the life-threatening hemolytic-uremic syndrome (HUS), which is the main cause of acute renal failure in children [9]. Infections in humans occur mainly through the consumption of food products and water contaminated with feces containing STEC strains. Domestic animals, mainly livestock, are the main source of these pathogenic bacteria [10]. They play an important role in the epidemiology of these colibacillosis because they are usually asymptomatic, thus their infections are not diagnosed, and consequent prophylaxis measures are not carried out.

Domestic ruminants may act as a source of pathogenic *E. coli* for wildlife too. In particular, they can contaminate, with their feces, pasture areas shared by wild animals. Deer are known as asymptomatic carriers of STEC strains because, similar to cattle, sheep, and goats, they lack vascular receptors for the Shiga toxins [11,12]. Other investigations have shown the spreading of STEC and other pathogenic *E. coli* strains among wild birds [13] and mammals [3]. Foxes have been supposed to be involved in the epidemiology of these infections too [3,14]; however, data about the spreading pathogenic *E. coli* in red foxes are generally scanty and lacking if referred to Italian areas.

This is a retrospective study carried out on collected DNA from feces of red foxes (*Vulpes vulpes*), with the aim to investigate the occurrence of *E. coli* genes coding the virulence factors intimin, hemolysin, and Shiga toxins in order to verify the potential role of red foxes as a source of STEC, EHEC, and EPEC strains.

## 2. Materials and Methods

Molecular analyses were carried out on a total of 109 DNA samples previously extracted from fecal specimens collected from red foxes. The animals were regularly hunted during 2016–2018 in central Italy and feces were collected for other investigations. No data about age and gender of the animals were available; furthermore, no gross lesions were observed during necropsies when feces were collected.

DNA was extracted from about 25 mg of each fecal sample with the commercial kit Tissue Genomic DNA Extraction Kit (Fisher Molecular Biology, Trevose, PA, USA), following the manufacturers’ guidelines, and stored at −20 °C. Single PCR protocols were performed to detect the following genes: *stx1* encoding Shiga toxins 1, *stx2* encoding Shiga toxins 2, *eaeA* encoding intimin, *hlyA* encoding hemolysin. Target genes, primers, and PCR conditions, previously described by Paton and Paton [15] (1998), are summarized in Table 1.

PCR amplifications were performed in a volume of 50 μL consisting of 25 μL of EconoTaq PLUS 2× Master Mix (Lucigen Corporation, Middleton, WI, USA), 0.5 μM of each primer, 3 μL of DNA and distilled water to reach the final volume, using the automated thermal cycler Gene-Amp PCR System 2700 (Perkin Elmer, Norwalk, CT, USA).

For each amplification, sterile water instead of DNA was included as negative control and Quantitative Genomic DNA from O157:H7 *E. coli* (ATCC^®^ 43895DQ TM) as positive control. PCR products were analyzed by electrophoresis on 1.5% agarose gel at 100 V for 45 min using PCR Sizer 100 bp DNA Ladder (Norgen Biotek, Thorold, ON, Canada) as DNA marker.

## 3. Results and Discussion

Among the 109 analyzed fecal samples, 31 (28.4%) were positive for at least one investigated virulence gene. In details, *eaeA* gene was detected in 21 (19.2%) samples, *hlyA* in 10 (9.1%), *stx1* in 6 (5.5%) and *stx2* in 4 (3.6%). Nine DNA samples tested positive for two or three virulence genes: five (4.6%) samples were positive for *eaeA* and *hlyA* genes, two (1.8%) for *eaeA* and *stx1*, one (0.9%) for *hlyA* and *stx1*, one (0.9%) for *eaeA*, *hlyA* and *stx2* (Table 2).

The detection of genes encoding intimin, hemolysin, and Shiga-toxins of *E. coli* in DNA extracted from feces of red foxes suggests that these animals are involved in the epidemiology of infections caused by pathogenic *E. coli* strains. This is a retrospective study carried out on DNA samples previously collected and stored in our laboratories. Consequently, it was not possible to isolate *E. coli* and determine if each fox harbored one or more bacterial strains.

Twenty-two samples had only one virulence gene, thus it is supposable that the 22 foxes harbored only one pathogen *E. coli* strain. In particular, 13 red foxes, in which *eaeA* gene was detected, probably harbored EPEC strains, and 6, in which *stx1* and *stx2* genes were found, probably had STEC strains. In feces of three red foxes, only *hlyA* gene was detected, thus it was not possible to relate this finding to a potential pathotype. Five samples had both *eaeA* and *hlya* genes, thus it could be supposed that the five foxes harbored EPEC strains; similarly one sample was positive for both *hlyA* and *stx1*, thus the corresponding fox could have a STEC strain in its intestinal tract. Two fecal samples had both *eaeA* and *stx1* genes; the two foxes could harbor EHEC strains if both genes belonged to the same *E. coli* isolate, but it cannot be excluded that they carried one EPEC and one STEC. Moreover, the sample positive for *eaeA*, *hlyA*, and *stx2* genes could correspond to a single EHEC strain or to different *E coli* isolates.

Among the obtained results, the detection of *stx1* or *stx2* genes, in a total of 10 (9.1%) animals, is the most relevant finding, because it suggests that STEC strains are circulating among red foxes. Data about pathogenic *E. coli* in foxes are very limited, thus our results are difficult to compare to other epidemiological scenario. However, the detected prevalence was quite similar to the value (8%) found in red foxes in Portugal [3]. A previous study carried out in northwestern Spain found a lower prevalence, in fact it recovered STEC in 5 (1.9%) among the 260 tested red foxes; moreover, 3 strains were classified as EHEC harboring *eaeA* gene, too [14].

The comparison of these investigations with others regarding the circulation of different *E. coli* pathotypes in wildlife highlights that red foxes are not reservoirs of pathogenic strains, mainly STEC and EHEC, as relevant as wild ungulates. In fact, STEC was found with a prevalence of 19.9%, 21.65% and 37% in red deer (*Cervus elaphus*) in northern Italy [16], Poland [17], and Portugal [3], respectively. Roe deer (*Capreolus capreolus*) were largely involved in the spreading of this pathotype too, with prevalence of 24.63% in Poland [17] and 72.6% in Germany [18]. Furthermore, STEC and attaching and effacing (AE)-STEC were identified in 13.83% of fallow deer (*Dama dama)* in Poland [19]. High percentages of wild boars (*Sus scrofa*) harboring STEC were also detected, with values ranging between 8% in Spain [20] and 28.29% in Poland [21].

Even though red foxes harbor STEC and other pathogenic *E. coli* less frequently than other wild animals species, they may represent a severe threat because in the last years they have a large spread in several European countries, including Italy, often invading human settlements in rural and urban areas. In this case, foxes harboring pathogenic *E. coli* can excrete the bacteria in environment shared by humans and their pets and consequently become a source of infection for them.

## 4. Conclusions

Red foxes seem to be involved in the epidemiology of pathogenic *E. coli*, including STEC strains. Further studies are necessary to better characterize *E. coli* harbored in the intestinal tract of red foxes also to verify the pathogenicity of these strains for them. However, even if asymptomatic, foxes may contaminate different environment and consequently represent a serious risk of infection for other wild animals, as well as domestic animals and humans.

In addition, *E. coli* are known to be able to carry virulence genes and share genetic information with its own and other bacterial species via horizontal gene transfer. Wild animals have been supposed to act as reservoirs of these virulence genes that aid pathogenesis but also as potential melting pots for novel gene combinations that could be more harmful to humans [22]. Therefore, investigations to verify the spreading of pathogenic *E. coli* in wildlife is pivotal in a One Health perspective.

## Figures and Tables

**Table 1:** Primers, investigated virulence genes, and expected amplicons of the PCR assays.

Target Gene	Primers	Amplicon (bp)	PCR Conditions
*stx1*	stx1F (5′-ATAAATCGCCATTCGTTGACTAC-3′) stx1R (5′-GAACGCCCACTGAGATCATC-3′)	180	35 cycles: denaturation at 95 °C for 1 min, annealing at 60 °C for 2 min, extension at 72 °C for 1 min
*stx2*	stx2F (5′-GGCACTGTCTGAAACTGCTCC-3′) stx2R (5′-TCGCCAGTTATCTGACATTCTG-3′)	255	
*eaeA*	eaeAF (5′-GACCCGGCACAAGCATAAGC-3′) eaeAR (5′-CCACCTGCAGCAACAAGAGG-3′)	384	
*hlyA*	hlyAF (5′-GCATCATCAAGCGTACGTTCC-3′) hlyAR (5′-AATGAGCCAAGCTGGTTAAGCT-3′)	534	

**Table 2:** DNA samples resulted positive for one or more virulence genes.

Virulence Genes	Possible Pathotype	N. Positive Samples (%)
*eaeA*	EPEC	13 (11.9)
*hlyA*	nc	3 (2.7)
*stx1*	STEC	3 (2.7)
*stx2*	STEC	3 (2.7)
*eaeA* + *hlyA*	EPEC	5 (4.6)
*eaeA + stx1*	EHEC	2 (1.8)
*hlyA + stx1*	STEC	1 (0.9)
*eaeA + hlyA + stx2*	EHEC	1 (0.9)

## Data Availability

The data presented in this study are available within the article.
